# ‘It’s a nightmare’: informed consent in paediatric genome-wide sequencing. A qualitative expert interview study from Germany and Switzerland

**DOI:** 10.1038/s41431-023-01468-9

**Published:** 2023-09-29

**Authors:** Johanna Eichinger, Bettina Zimmermann, Bernice Elger, Stuart McLennan, Isabel Filges, Insa Koné

**Affiliations:** 1 Institute for Biomedical Ethics, University of Basel, Basel, Switzerland; 2 Institute of History and Ethics in Medicine, TUM School of Medicine, Technical University of Munich, Munich, Germany; 3 Institute of Philosophy & Multidisciplinary Center for Infectious Diseases, University of Bern, Bern, Switzerland; 4 Center for Legal Medicine (CURML), University of Geneva, Geneva, Switzerland; 5 Medical Genetics, Institute of Medical Genetics and Pathology, University Hospital Basel and University of Basel, Basel, Switzerland; 6 Department of Clinical Research, University Hospital Basel and University of Basel, Basel, Switzerland

**Keywords:** Ethics, Paediatrics, Medical ethics, Patient education

## Abstract

The use of genome-wide sequencing (GWS) in paediatrics has added complexity to informed consent (IC) and pretest counselling because of the vast number and interpretation of potential findings, and their implications. However, empirical data from continental Europe on these issues remains limited. This study therefore aimed to explore the experiences and views of medical geneticists working with children in Germany and Switzerland regarding the challenges of obtaining valid IC in paediatric GWS. Qualitative interviews with 20 medical geneticists were analysed employing reflexive thematic analysis. In the interviews, many medical geneticists questioned the validity of parents’ IC due to the enormous amount of relevant information given and the variety and complexity of the possible test outcomes. Key barriers identified included familial implications, administrative challenges and struggles with non-directiveness. Medical geneticists’ suggestions for improvement included increasing the number of genetics professionals and better information material, which is crucial as GWS becomes a diagnostic standard in the early care pathways of children. An adjustment of aspirations from still existing ideal of traditional fully IC to appropriate IC seems to be needed. Such a more realistic and ethically sound adaptation of the requirements for IC can lead to better ‘informedness’ and improve the validity of the consent. This might also help reduce the moral distress for the medical geneticists involved.

## Introduction

Genome-wide sequencing (GWS) (whole-genome sequencing, whole-exome sequencing) is now routine in paediatric clinical care and research in many countries [1]. It has demonstrated clinical utility, especially to help children with undiagnosed genetic diseases [2] and has shown a high diagnostic yield [3, 4]. One of the several new layers of complexity that GWS has added to medical genetics and also to paediatric medicine—notwithstanding its many benefits [5–7]—concerns informed consent (IC) and pretest counselling.

IC is a legal and ethical requirement to respect patients´ autonomous choices [8], prevent harm [9], and promote trust [10]. Ethically valid IC should involve more than formal acts such as the provision of information material and collection of signatures [8]. Valid IC requires that the person providing the consent (1) is competent, (2) has sufficient and appropriate information and understands it, and (3) makes the decision freely and without coercion [11]. In the context of genomic sequencing—particularly with children [12]—the idea of fully IC has been called into question because of GWS’s complexity due to the vast number of potential findings, implications, and consequences [13–16]. It encompasses not only understanding the possibility of a clear diagnosis, but also the possibility of obtaining no result explaining the cause of a condition (as to our current knowledge, which may change over time) and hence cannot exclude a genetic cause, but also potential results of yet uncertain clinical significance, results of a probabilistic nature and unsolicited findings. As a consequence of these challenges, various alternative consent models have been proposed such as forms of broad consent [13] or the tiered-layered-staged model of consent [17]. These are based on the recognition that it is not possible, nor ethically necessary, to disclose or provide the complex information in all its granularities, as information overload can thwart understanding, autonomous decision-making, and hence valid IC [18]. It has been suggested that a basic set of essential information should be guaranteed for all patients and/or their caregivers (herein called ‘parents’) [8]; but beyond that, the amount of information should be tailored to the specific needs and preferences of individuals.

In Switzerland and Germany, GWS is used solely for diagnostic purposes; purely predictive tests are not permitted in paediatrics. Guidelines in both countries make the following distinction for counselling for genetic testing: For diagnostic testing, pretest counselling is a requirement and genetic counselling must be offered as an option. Genetic counselling is legally mandatory for presymptomatic and prenatal testing or genetic testing for the purpose of family planning (§10 Abs. 2 GenDG; GUMG Art.21).

A controversial aspect regarding IC and pretest genetic counselling interactions is non-directiveness and the extent to which it is appropriate to guide the decision-making of patients (or their parents) and make recommendations [19]. Non-directiveness has long been upheld as a principle in medical genetics and genetic counselling [20, 21]. However, the principle has increasingly come under pressure [22] and has been removed from some guidelines [15, 19] because of advances in medical genetics for establishing diagnoses, allowing specific treatment and surveillance recommendations as well as preventive measures [21]. Nevertheless, the practical guidelines in Germany and the Swiss legal framework uphold that genetic counselling should be non-directive [23–27].

Although several theoretical efforts have been made in recent years on how to better achieve valid IC in genomic sequencing, there are limited empirical studies that examine how health care professionals who obtain IC in practice perceive the process and the challenges involved [15, 28]. This is particularly the case when it comes to the paediatric clinical context [15]. The existing research primarily comes from Anglo-American countries (such as [29]) with very limited research involving continental European participants. This study, therefore, aimed to explore the experiences and views of medical geneticists working with children in Germany and Switzerland regarding challenges in obtaining valid IC in paediatric GWS.

## Materials and methods

For a comprehensive methods section see Supplementary 1.

### Study design

Participants were primarily selected through purposive sampling combined with snowball sampling [29]. We included medical geneticists from two major continental European countries (Germany and Switzerland). In both countries, medical geneticists are board certified physicians and are typically responsible for obtaining IC for paediatric GWS (genetic counsellors, for example, are not yet a recognised profession). We interviewed 20 medical geneticists who work with children in Germany (*n* = 10) and in Switzerland (*n* = 10) (German-speaking part and Romandy); 15 worked in academic hospitals and 5 in private specialty practices or private laboratories. Interviews were conducted between February 2020 and April 2021. An interview guideline was developed based on available literature to explore medical geneticists’ views about the ethical issues in paediatric GWS (Supplementary 2). All interviews were audio-recorded and had a mean duration of 55 min (range 29–71 min). They were transcribed verbatim, and transcripts were pseudonymised. We adopted a pragmatic approach in determining data saturation and critically evaluated theme saturation throughout the data analysis phase [30].

### Data analysis

Using the interview transcriptions in their original language, data were analysed inductively by JE and IK using the qualitative software MAXQDA and employing reflexive thematic analysis [31]. A coding system encompassing 133 codes was developed by comparing and discussing individually developed codes, coded segments, and writing memos. In an iterative and interpretive process, main themes on the overall topic of IC were generated and critically reflected and discussed with the other co-authors based on analytic reports written by JE.

## Results

**Fig. 1:**
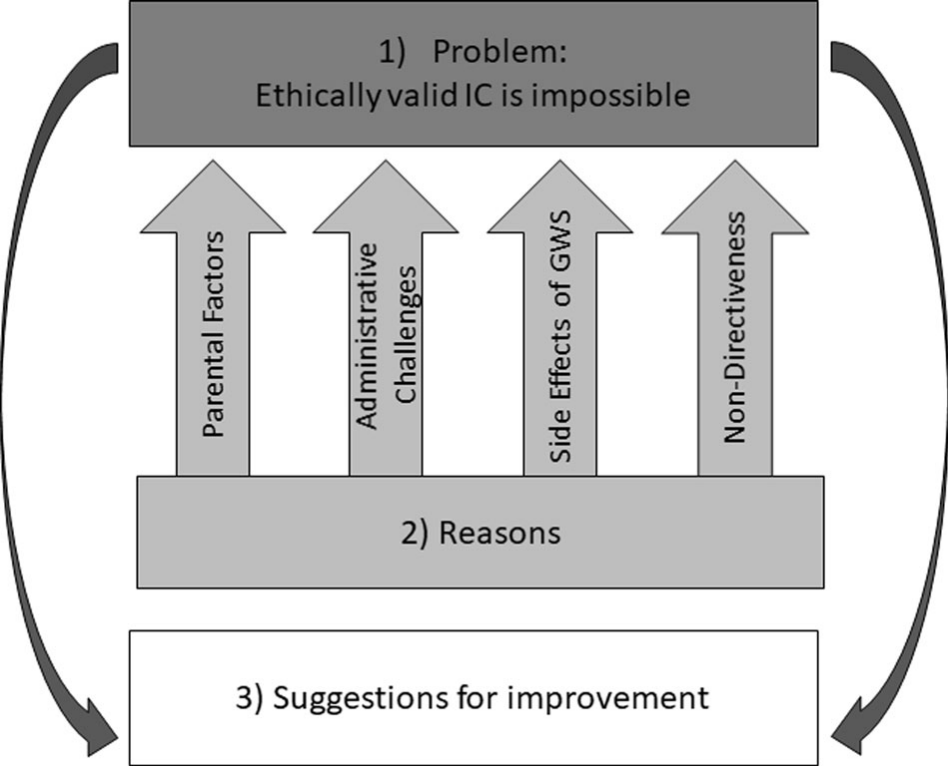
‘The three themes’. ‘Fig. 1 illustrates the three main themes we identified in the qualitative analysis of the interview data regarding informed consent in paediatric GWS’.

We developed three themes: First, we established that several interviewees perceived it as impossible to obtain ethically valid IC in the context of paediatric GWS. Second, we present reasons for this impossibility mentioned by participants. Third, we summarise participants’ suggestions for improvement (Fig. 1).

### The impossibility of obtaining ethically valid IC in paediatric genome-wide sequencing

Several participants expressed doubts that ethically valid IC was feasible in the context of paediatric GWS, given its great complexity and the sheer endless number and range of different possible outcomes and consequences (see Table 1, Q1). Although some participants found this unproblematic, noting similar situations where one has to trust experts (for example, going to the bank for complex financial matters), others considered the inability to obtain valid IC highly problematic, as the lack of understanding could lead to a wrong decision that harmed and burdened parents afterwards (Q2). Some participants also expressed uncertainty as to whether they could assess whether the content had really been understood (Q3) and perceived a risk that understanding would fade away shortly after counselling. Several participants emphasised the parents’ emotional coherence as an essential factor of valid IC and the relevance of the parents’ fundamental values, both in deciding for and against GWS and learning about unsolicited findings (UFs)(Q4). According to them, IC was valid when it corresponded to the parents’ basic values and gut feeling, even if it was impossible to understand all the implications and complexity of GWS.

**Table 1:** Quotes supporting the results.

THEME	SUBTHEME	CODE	EXAMPLE QUOTES
1. The impossibility of obtaining ethically valid IC in paediatric genome-wide sequencing		*Q1*	‘*It’ s a nightmare, the informed consent. […] To explain all these things, you know, lengthily and broadly beforehand, is simply an impossibility. Simply an impossibility.*’ *CH7*
		*Q2*	*‘That they find something which the patient perhaps did not really want to know, something that simply because he could not make a better decision at that moment, then accidentally ticked ‘yes’ for the incidental findings, which then he actually did not really want to have. Yes, that the patients give too much consent too early, so to speak, and are then overwhelmed with the result.’ DE12*
		*Q3*	*‘Of course it varies, but, in general, I have the feeling that they understand it well. But whether that is really the case is, of course, a completely different question. And you really do experience many situations where, for some reason, you have contact again later and then realise that a lot has been misunderstood. It’s difficult to assess that.’ DE17*
		*Q4*	*‘So I do not think anyone can oversee that to such an extent. […] But I think people may already have a bit of an inkling of what makes them uncomfortable and what does not. I think it also goes to an emotional level. There are patients who sit down ‘You know, I want to know everything anyway’. That’s their basic attitude anyway. And then you notice that there are people for whom this is scary, who also say ‘Oh, I do not know, I do not think this changes anything for my child and somehow it is all too much for me’. So I have the impression that they can make a valid decision for themselves.’ CH2*
2. Barriers undermining valid IC	Parental factors	Q5	*‘I think it depends on the intelligence of the person seeking advice. We really do have extremely differentiated patients who I think understand very, very well what they are getting into. And then of course there are also a lot of worse patients who understand it very badly.’ DE13*
		*Q6*	*‘And I think with distinguished people who speak good German, this could also be understood. But there are people who are not at all interested in what we do. And also have no interest at all in understanding it.’ DE20*
		*Q7*	*‘Then, you have the parents, they anyway ask if it can be useful to the child. And of course, it can be useful in terms for example in epilepsy, we know that some genes are associated with a better answer to that or that medication. Or that sometimes we can find things that you need to follow. So this, we say, they always ask about if we can change it. We explain that we cannot change the gene. So, of course the more the parents are/ the family life is struggling with the troubles of the child, the more there is a search for a solution that could be brought by the genetic testing. So, during the consultation it is always/ we have to explain what could be brought, but we also tell the parents that we cannot find everything. Because there is a strong hope when we say that we could find this or that, there is strong hope that we will find.’ CH10*
		*Q8*	*‘So, there is this, I do not know if it’s magical or pseudo-religious or, I do not know, guilt or punishment from God or whatever, it’s actually not that rare. Self-blame is also an issue. The child has a developmental disorder, it was because I drank half a glass of wine on New Year’s Eve, or had so much stress, or was ill, or fell on my belly, or whatever. Sometimes there are really things and ideas in people’s heads where you can perhaps reach them on an intellectual level. That they understand. But I believe that these ideas that have developed over the years, which explain the cause of a diagnosis, cannot be disproved with a one-off counselling session.’ DE18*
	Administrative challenges	*Q9*	*‘I think that, in an ideal world, there would be more time and personnel to give a better information prior to consent. I think as in the Swiss insurance system, and how people are/ how hospitals are financed, it is very difficult to get the optimal consent.’ CH6*
		*Q10*	*‘And even when it is a diagnostic request, the individual laboratories handle it very differently. And that is a very big difficulty. There is no consensus among the testing laboratories in this regard. And it can’t always be taken from the respective forms or declarations of consent, and sometimes it also changes during the time when you have something analysed. Because the analyses now also take longer than 3 days. […] I want to inform the parents and the family about what is being done. And not to have done anything else afterwards than what I had explained.’ DE17*
	Side effects of GWS	*Q11*	*‘The incidental findings is more difficult. I will give you an example we had: A patient that we saw for developmental delay, and he was found to be a carrier of a recessive cause for developmental delay, but he was a carrier. But his parents were consanguineous, so then we had to go back and explain that incidental finding to them and that we were, even though it was not the cause of their child’s developmental delay, for their future reproductive risk we would recommend testing both parents and their other children might also be carriers and this, so it would be something for their grandchildren. And this level of incidental finding is somehow difficult to get into at the first consultation. So I say not every possibility for incidental finding can be addressed in a first consultation that would be a level of information that would overwhelm most parents. And would cause you to have a consent that takes two hours.’ CH6*
		*Q12*	*‘I think we have to consider who has the right to know and not to know, and who is ultimately affected. I think it is legitimate that parents do not want to know any incidental findings for their child. But if, by chance, we see a finding that has an acute therapeutic option for the child, in childhood, not in adulthood, in childhood, I see an obligation to disclose that. It’s also simply because you examine so much during these examinations that you cannot discuss absolutely every single situation in the pretest counselling. And I believe, and this is a principle that applies to the whole of medicine, that in a situation where it is unclear, the patient’s well-being must be kept in mind. And in that sense, at that moment, I would say the principle of the patient’s well-being takes precedence over not knowing. Because the patient is the child, who cannot express himself. […] It is a concrete therapy option. I mean, it would be similar to a CT scan for a patient who has abdominal pain. And now you see that the patient has a lung tumour. Or a white shadow. And he said before that he did not want any incidental findings. Do you now not tell him that he has a lung tumour and wait 3 years until he dies from it? I do not think you can do that. I think you have a medical obligation when you see a finding that needs treatment to discuss it.’ CH2*
		*Q13*	*‘But the problem is that every now and then you find things, for example, let’s say it’s a boy. And there, by chance, you find a breast mutation, a mutation in a breast cancer gene, right? A clear pathogenic mutation. He could have gotten that from his mother, of course, and that could have relevance for the mother. At the moment, isn’t it? And all that, you just have to communicate that clearly beforehand, because otherwise you are then, in French you say embêté, you are then, you are then unwell. […] And that’s, you see, it’s so complex, isn’t it? So it’s, genetic counselling is the be-all and end-all of these whole genomes, whole exome sequencings.’ CH7*
	Requirement of Non-directiveness	Q14	*‘The less intelligent someone is, the more I can leave them out in the cold if I leave everything to them to decide. So when I really have the feeling that they are very simple people, I tend to behave in a more directive way. As I think it would be best for them then. People might accuse me of that, but I think that in these cases I am no longer non-directive.’ DE17*
		Q15	*‘I have to try to show all the options. I have to set out the legal-moral framework, so to speak, yes? I just have to say this and this and this. Those in your situation can do this, this, this, this, all that. And then they usually look at me like a car. Very few of them have a concrete idea beforehand. […] The problem I see is that most people simply have too little knowledge for the true non-directiveness. So many people have to be guided in some way, so to speak. Yes, and just tell them by saying ‘Most others in your situation would probably choose this path’. Yes, well, most people are helpless with just information about what they can do.’ DE12*
		Q16	*‘So there, for example, I think that even in genetics, non-directiveness comes to an end, because it’s about more than just knowledge. I actually believe that this is one of the points why we are so non-directive, why we are so committed to it, because for a very long time we did not provide any therapeutic consequences. But fortunately that is changing. And with the tumour issues, things have changed enormously, so I think this non-directive approach has come to an end.’ DE11*
		Q17	*‘It is problematic. Because you decide a bit for others, which actually shouldn’t be the case. But then they are often hopelessly overburdened by the situation.’ DE16*
		Q18	*‘But I think when it comes to actually having a diagnosis so that you can actively influence a patient’s therapy, I think the medical principle applies. That you can make a recommendation, a medical recommendation. […] So this is not just about counselling, this is a co-assessment of a patient from a clinical-diagnostic background. It’s like when you go to the family doctor because you have abdominal pain and you want to know what the cause is for your abdominal pain. And that’s the same: you have symptoms that you cannot classify and you want to know the cause. And that is a medical-diagnostic process. Of course, it can have various other implications, but even if the doctor does an ultrasound, he could or could not find the cause.’ CH2*
		Q19	*‘So I think we are now talking about diagnostic consultations. Um, and here I am actually of the opinion that they can always proceed in the same way in terms of non-directiveness, and should actually always be non-directive.’ DE19*
3. Suggestions for improving the IC process	Training and experience of the physician obtaining consent	Q20	*‘I think the understanding depends on the presentation of the problems. Of how good a teacher the genetic counsellor is. I find that one should not give a lecture of genome analysis to the parents. Keeping it quite simple. And there are ways to be simple, and I would say most of the parents, a good fraction of the parents understand.’ CH9*
		Q21	*‘You have to provide everything that you legally and professionally need. And there I have to put myself on the level of the parents according to their intellectual abilities. It won’t work without that. And I cannot say, because maybe he doesn’t understand me or this family doesn’t understand me, then I will ust do it, they will sign it somewhere. So […] because I think we have the verbal means to get to this level and thus I have to take the time.‘ CH1*
		Q22	*‘I try to explain it as much as possible in a figurative language. So, I always talk about a library with many thousands of books on shelves. And I tell the families to imagine the shelves as the chromosomes. And the books as the genes, or manuals on these shelves, which then contain text that the body needs to fulfil its function. And then I say, the chromosome analysis, just look: Are the shelves there and are there any big parts missing from the shelves? The chromosome microarray analysis, I say, is practically an inventory of the library, where just every single book is looked at: Is it there, are there an appropriate number of books present, are any missing or extra? And with exome sequencing, I say: Okay, and now it’s a completely different kind of examination, because now the books are opened and we look in the text to see if there are any typing errors.’ DE19*
	Administrative changes	Q23	*‘I think that the problem is that they are asked to decide when they are not really ready. They have to decide within most often/ or they think they have to decide within one hour, because it is the time frame of the consultation. And I think it is too fast. And they cannot really realise, discuss with the wife or husband and take their time to think about it. So, for me in most of our consultation it is a problem. […] As they come with the child, then often is complicated because there is school absence, and the mom or the dad had to take of the work. I think they also feel the pressure of whether it is better to be fast and decide now. So even if I say ‘You can change your mind. You can call me back’, I have the feeling that it is an ongoing process and they think it is better to do it that way.’ CH3*
		Q24	*‘Because what I find particularly difficult is that the families are confronted with these issues and questions during the consultation and cannot really prepare themselves. So they are faced with some serious decisions that I ask them to make. And often within a few minutes, they have to tick the box ‘yes or no’. Therefore, what we want to do is, we will now create educational videos on these issues over the next year, and we will send the families the links in advance. So that they can already have a look at this, this information. Or if necessary, they can watch it two or three times. And they can also deal with two or three of these key questions, including the topic of unsolicited findings, at home. And then come to us for the consultation prepared with their questions.’ DE19*
		Q25	*‘Visiting people at home and collecting their pedigree and so on. That’s something you cannot do as a doctor alone, and it’s also the case that there is quite a distance between doctor and patient, and a counselor is a bit closer to the people and more at eye level compared to a doctor.’ DE16*

### Barriers undermining ethically valid IC

#### Parental factors

Participants regularly identified the parents’ comprehension as a key issue that undermined the IC process (Q5). It was noted that this was largely due to educational and language barriers. Although interpreters were provided, they often lacked genetic expertise and specific terminology. Some participants stated that several parents due to the feeling of being overwhelmed had no interest in understanding and exercising IC (Q6). Furthermore, participants reported that many parents had unrealistic expectations and *‘false hopes’* (CH1) regarding GWS. They had the impression that parents often assumed that GWS would always lead to a specific diagnosis, open up new treatment options or even cure the child’s disease. These unrealistic expectations hampered parents´ understanding, especially in families that struggled to come to terms with their child’s disease (Q7). Participants emphasised the importance of clearly discussing the limits of GWS in the pretest information session, including the possibility of variants of unknown significance or of no result at all due to the current state of knowledge. These limits were, however, often perceived to be difficult to understand for parents. Nevertheless, participants noted that GWS could still add value despite its limitations, e.g. ending the diagnostic odyssey, putting the child’s condition into perspective and adapting medical follow-ups and treatment, relieving parental feelings of guilt, or obtaining a name for the child’s disease.

It was also reported that parental beliefs could occasionally cloud understanding and decision-making processes: these could be religious attitudes, but also other long-held beliefs, such as that the mother is to blame for the child’s developmental disorder because she *‘drank half a glass of wine on New Year’s Eve’* (Q8).

#### Administrative challenges

Participants also frequently identified insufficient time for counselling and limited personal and financial resources as issues that undermined the IC process (Q9). Some stated that due to a lack of time, it was not even possible to discuss all the items on the consent form that parents had to sign. According to their perception, especially for parents who were *‘very uncritical’* (DE13) the pretest counselling was carried out less carefully.

Another challenge identified affecting the issue of valid IC related to some laboratories’ volatile and inconsistent reporting practices. A German participant mentioned that agreements between parents and geneticists resulting in IC were sometimes violated because laboratories did not adapt their report to the parents’ consent (e.g. regarding the reporting of UFs), the reporting standards of the laboratories were different, or because their reporting policy had changed in the meantime (Q10).

#### Side effects of GWS

Potential UFs were often seen by participants as the most complicated and ethically sensitive issue to discuss (Q11). It was reported that the discussion about UFs often absorbed a lot of the time and parents always had to make one or more concrete decisions regarding them, although the options were reported to vary depending on the department and participants involved. Even if parents had previously decided in the IC that they would not have wanted to know UFs, some participants stated that in case important actionable UFs emerge, they would overrule the IC and communicate the findings to the parents (Q12), since according to their interpretation, the principle of beneficence held greater importance than the principles of autonomy or the right not to know in these particular situations.

The complexity of the IC process was increased by the potential implications of an outcome for the whole family, which further complicated the understanding of the possible consequences of an analysis. Discussing all these scenarios in pretest counselling was impossible according to several participants. For example, certain results—especially when trio analyses had been conducted—suggested that other family members were also affected, which might be of immediate health concern to them. In addition, some analyses revealed cases of misattributed paternity; however, prior information about this was usually only indirect. Due to these familial implications, there is a risk that the parents’ personal interests may conflict with those of their child, adding another complicating dimension to decision-making (Q13).

#### Requirement of non-directiveness

Participants described many parents as struggling with non-directiveness in the IC process (Q14)—concerning the question of whether GWS should be carried out at all and the question of whether UFs should be reported. Parents frequently asked them: ‘What would you do?’ Some participants emphasised that they never answered this question to follow the principle of non-directiveness. Others said that although they would prefer not to answer, if the parents were *‘very helpless […] either intellectually, or language-wise, or under time pressure’* (DE20), they did. Other participants chose to offer guidance by providing other parents’ choices as an example. Several participants draw a direct link between not being (fully) non-directive and parents being overwhelmed by the complexity of GWS (Q15). Some considered a more directive attitude appropriate and part of their role as a physician (Q16), while others considered it problematic that they then decided for the parents (Q17).

Additionally, participants showed differing interpretations of the legal requirements: Some participants differentiated between formal, detailed genetic counselling and pretest-counselling (required for any medical procedure, including diagnostic GWS), which yield different requirements and standards, e.g. regarding the requirement of non-directiveness or the possibility of giving clear recommendations (Q18). Other participants did not make the same distinction or emphasised that in their view, the requirement of non-directiveness also applied to diagnostic pretest counselling (Q19).

### Suggestions for improving the IC process

Participants saw a strong need for optimisation to improve the GWS consent processes. Even those participants, who said that IC in paediatric GWS was essentially problematic emphasised that the ideal could nevertheless be approached more closely by improving the professional skills of the person obtaining consent and tackling administrative changes.

#### Training and experience of the physician obtaining consent

The communication and teaching skills of the medical geneticists were seen by many participants as the key factor allowing parents to understand the relevant information about GWS and to obtain IC (Q20). They learned how to conduct these discussions through communication training and practical experience during their residency training. This communication flexibility and the choice of appropriate verbal means were essential to avoid being too paternalistic and deciding too quickly for the parents (Q21). In most cases, the wealth of information had to be reduced and explanations would have had to be kept simple in terms of language to avoid overwhelming the parents. Participants also reported the analogies they used to make what they explained more accessible (Q22). For Germany, it was highlighted as a particular problem that other medical specialists could also request GWS in the case of diagnostic testing, which repeatedly led to parents *‘not being informed properly’* (DE18).

#### Administrative changes

Several participants emphasised that it was sometimes important to extend decision-making beyond the pretest consultation and be available to parents afterwards, allowing parents time for their decision and possibly even encouraging a postponement of GWS if there was no medical urgency (Q23). Furthermore, participants mentioned improved supporting materials such as brochures or educational videos that parents could watch before, during, or after the consultation to provide parents support for dealing with important questions, e.g. regarding UFs (Q24). In addition, several tasks (e.g. taking blood samples, updating databases, collecting pedigrees, providing basic information to parents) could be reassigned to other clinical staff members to allow participants to dedicate more time to the IC process. Several participants emphasised that genetic counsellors could be of support for this, also because they might more easily establish a closer relationship with the parents (Q25).

## Discussion

This is one of the first studies to examine the views of medical geneticists about IC for GWS in continental Europe [14]. With its empirical insights into medical geneticists’ perceptions in the Swiss and German context, the study complements the existing international debates on IC and pretest counselling for GWS in a practice-oriented manner. Many medical geneticists in our interviews questioned the validity of the IC given by parents, as understanding was complicated due to the vast amount of information, as well as the variety and complexity of the possible outcomes. German and Swiss medical geneticists identified similar challenges as known from studies from Anglo-Saxon countries, which highlight that understanding of patients or their parents is a major challenge from the perspective of medical geneticists or counsellors [32]. A recent systematic review summarising the understanding of parents whose child was offered GWS also points out this difficulty [33]. The at times value-laden language of participants regarding the ability of parents to understand should not, however, overshadow the fact that the main responsibility lies with clinicians and the medical system to provide information in a way each patient can understand. Medical geneticists mentioned further factors that complicated a meaningful engagement with the parents during the IC process such as unrealistic parental expectations, inconsistent reporting practices by laboratories, and struggling with non-directiveness.

There was uncertainty among the interviewed medical geneticists as to what ethically valid IC in paediatric GWS should require. Many described IC as problematic, especially those who considered the traditional notion of fully IC as the benchmark. Beauchamp and Childress state in the ‘Principles of Biomedical Ethics’ that an improper ideal of fully IC would lead to unnecessary scepticism and was better replaced by a realistic account of adequately IC [8]. The recently published PROMICE framework (PROmoting Morally Important Consent Ends: PROMICE) [34] also underscores this by emphasising a move away from fully to *‘appropriately’* IC. The idea is that the moral standard against which the validity of IC is evaluated is the realisation and equilibration of four morally important goals: promoting wellbeing, respecting autonomy, promoting autonomy, and trust in medicine. This can help determine how the idea of IC can be meaningfully operationalised within the context of paediatric GWS. By having in mind that the goal should rather be an *appropriate* IC, it could also be prevented that the idea of IC is completely thrown overboard, because of the perceived unattainable ideal of addressing all aspects in the pretest counselling (then resulting in paternalism or superficial counselling). A realistic and ethically sound adaptation of the requirements for IC can lead to a better ‘informedness’ and validity of the consent given. It might also reduce the moral distress for the medical geneticists involved. Since its emergence in 1984 [35], the concept of moral distress has gained significant popularity in recent years to describe the discomfort that healthcare professionals experience when they are unable to fulfil their perceived responsibilities [36, 37]. The factors influencing participants’ positions on the spectrum between the unattainable goal of fully IC and that of appropriately IC, and whether they perceive this as problematic or not, likely encompass various components. These may include the expectations regarding the health literacy of parents, overall perspectives on the doctor-patient relationship, self-reflection, and training.

Our findings suggest that several medical geneticists applied a roughly layered, tiered IC model [17] by attempting to provide a standard set of knowledge to all parents but then tailoring all further information bundles to individual needs. Which concrete elements belong to this basic informational layer, which all parents should receive as a minimum, is the subject of various other studies [38, 39]. Some participants even implemented the ‘staged’ aspect by encouraging parents to defer the decision (or part of the decision) to a later time, or even by adapting the diagnostic steps to the current parents’ decision-making abilities. A temporal separation between obtaining initial consent for a diagnostic work-up and later, less high-stress discussions with clinical genetics services, where the question of unsolicited and secondary findings may be raised and explored under less pressure, is often practised in cases of acutely ill neonates or children in the NICU/PICU. However, such approaches necessitate careful coordination with the reporting laboratory.

The varied approaches taken by institutions and geneticists in managing unsolicited and secondary findings raise various questions. For example, parents might be presented with different options at one clinic compared to others. Therefore, it is crucial to ensure clear communication during pretest counselling about how such findings will be handled, especially in the case of ‘very actionable’ findings that will be communicated in any case.

In addition to specific content, our findings also reaffirm that an important part of pretest counselling is expectation management and the uncovering of expectations which cannot (always) be met using GWS [27, 40, 41]. Those include the expectation that the test will generate a definitive diagnosis (‘name of the disease’) in all cases or that it will definitively exclude a genetic cause. Pretest counselling should emphasise repeatedly the provisional nature of the results obtained, that there is the possibility of no results explaining the cause of the child’s condition or of results with uncertain significance regarding the cause of the condition, and that even if there is a causal mutation found and diagnosis identified, there may (currently) be no specific medicinal treatment available.

Our findings show that the interviewed participants have differing views on (a) whether non-directiveness is good or bad in principle, (b) what non-directiveness actually means, and (c) whether it is legally necessary. These differences did not depend on jurisdiction and were found in both countries. These uncertainties may not only lead to further moral distress among the involved medical geneticists, but may also harm the quality of the IC processes. Drawing on the moral goals described in the PROMICE framework that the IC process should fulfill, we argue that the principle of non-directiveness in general should not impose too great a burden on parents and medical geneticists. Keeping these goals in mind, it may well be ethically justified in some situations not to be non-directive. This is also in line with the contemporary literature on genetic counselling (e.g. [42]). Person-centredness, which is also called for in the guidelines of the German Society for Medical Genetics [43]—or in our case even family-centredness—does not necessarily require a purely non-directive stance. Especially in the context of diagnostic paediatric GWS, the described goal of promoting well-being could be given greater relevance than promoting autonomy, since it is proxy decision-making. Promoting autonomy in most cases of paediatric GWS primarily aims to preserve the autonomy of the proxy decision-makers, rather than that of the children themselves. Therefore, the goal to promote the child’s wellbeing takes on particular weight in the balancing of the two principles as it pertains directly to the child’s best interest. Here, however, the aim should not be, as some interviewees said in the case of supposedly ‘very helpless’ parents, to make a recommendation as one would act oneself or the majority of others, but always to assist the patient in choosing a course of action that accords with their values and the respective consequences acceptable for them. Also, the fact that in the diagnostic context, formal genetic counselling does not necessarily have to be provided should not be used as an excuse for not providing a sound IC process.

To improve the understanding and ‘informedness’ of parents, innovative information materials could make a useful contribution to supplement the personal pretest counselling interaction, as some of our interview partners explicitly desired. In particular, those that parents could watch in preparation for (or as a follow-up to) the actual discussion, to generally improve genomic literacy, to be able to sustainably deal with some important questions in advance, and not to be overwhelmed by the amount of information during the discussion [44]. This might also offer the opportunity to involve the children to a greater extent in the decisions and to discuss the matter with them. Purely text-based materials may be of limited value [45] and short video sequences seem to be a good alternative [46]. Other more sophisticated alternatives would be decision-aiding virtual reality scenarios or serious moral games [47] to better empathise with different decision-making scenarios and gain greater awareness of one’s own values. These could also be advantageous when discussing hypothetical scenarios during pretest counselling, which can be particularly challenging, as the actual implications of such potential outcomes are often difficult for parents/patients to imagine. These types of information materials are often more attractive, less reliant on the education of the recipient, and easily accessible on mobile devices. Furthermore, with relatively little effort, these materials could be captioned with voice-overs in different languages to also mitigate the translation problem frequently mentioned in the interviews. Once ready, they could well be distributed among other sites and clinics. Creating these types of supporting material is a genuine multidisciplinary task, potentially including genetics professionals, psychologists, ethicists, intercultural mediators, design specialists, etc. However, considering the ongoing struggle over financial resources in the healthcare system, the funding of such tools poses a challenge.

Reinforcing the high influence of resource constraints in the clinics and laboratories reported in studies from other countries [15], medical geneticists highlighted that insufficient time and personnel was a key issue impeding the IC process. Since the number of specialists in medical genetics is stagnating but the number of genetic tests requested is steadily increasing and the knowledge on genetic conditions and rare genetic diseases is expanding rapidly, an escalation of personnel shortage is to be expected. Thus, reflecting ongoing discussions in Switzerland and Germany, several study participants called for the widespread introduction of genetic counsellors also in German-speaking countries to further alleviate these shortages, under the condition that these would work under the professional responsibility of medical geneticists and would be qualified with a certified Master’s degree programme [48]. Trust in and acceptance of the work of genetic counsellors seems high among medical geneticists interviewed in this study. Both factors have been mentioned as important to pave the way for a successful introduction of these professionals in German-speaking countries [49]. However, providing financial resources for training and job opportunities will pose the same challenge as it does for medical geneticists.

Moreover, due to these financial and personnel resource constraints, reanalysis of the collected data and recontacting patients proactively was deemed unfeasible by our participants and thus received little attention in the IC discussions. The study has several limitations. Our sample size does not allow for quantification or weighing of different views and experiences. Instead, with this qualitative approach, we attempted to address a variety of perspectives and assumed theoretical saturation in the attitudes expressed by the participants after the 20 interviews conducted. In addition, medical geneticists agreeing to participate in our study might more likely have a higher sensitivity to ethical challenges and question the processes. While we acknowledge that the results could be biased in this respect, it is still helpful to improve clinical practice recommendations by highlighting critical issues. Assumptions about the parents’ educational background were repeatedly brought up in the interviews. We interpreted those as a subjective perception of our interview partners, which might be biased by language differences, cultural background etc.

This study shows several challenges medical geneticists in Germany and Switzerland perceive when obtaining IC in paediatric GWS. It particularly illustrates that IC is questioned by several medical geneticists, which seems to be linked to still existing demands of an idealised, traditional fully IC. An adjustment of aspirations from fully to appropriate IC is needed in this context. It also became apparent that there may be a need to put the principle of non-directiveness into perspective, especially in the diagnostic setting in paediatrics. Finally, there is a need for improved information materials and for increasing the number of genetics professionals in these two countries. Further research would be relevant to enquire patients’ perspectives on the IC process as GWS becomes a diagnostic standard in the early care pathways of children and mainstreaming of genomics is ongoing.

### Supplementary information

Supplement 1

Supplement 2

## Data Availability

The data that support the findings of this study are available from the corresponding author upon reasonable request but cannot be made publicly available.
