# Supplementary material for: ‘It’s a nightmare’: informed consent in paediatric genome-wide sequencing. A qualitative expert interview study from Germany and Switzerland
Source: Eur J Hum Genet. 2023 Sep 29;31(12):1398–406. doi: 10.1038/s41431-023-01468-9 (PMC10689462; doi:10.1038/s41431-023-01468-9)
Supplement: Supplementary file 1 — Supplement 1 [file 41431_2023_1468_MOESM1_ESM.docx]

### Supplement 1 Extended Methods Section

This paper is part of a PhD project regarding ethical considerations in paediatric genome-wide sequencing including semi-structured interviews with clinical geneticists working with children. Here we report the results of one major topic area that we found to be important within the interviews, namely informed consent (other topics will be published elsewhere).

Study documents were reviewed by the responsible ethics committee (Ethikkommission Nordwest- und Zentralschweiz; EKNZ). The EKNZ declared that the study does not fall under the Swiss Human Research Act (Art. 2) because no health-related data were collected and data were coded and pseudonymised. Also, interviewing health professionals does not require ethical approval in Switzerland. Hence, ethical approval was not needed. Nevertheless, the EKNZ issued a declaration of no objection (Req-2019-00467) confirming that the project fulfills the scientific and ethical standards for research with humans (Art. 51, Swiss Human Research Act). Written informed consent was obtained prior to data collection and was considered as a subject to renegotiating over time. An information sheet was provided explaining the nature of the project, including the right to withdraw or stop the interview at any moment. The methods of the study are presented in accordance with the Consolidated Criteria for Reporting Qualitative Research (COREQ) reporting guideline (1).

### **Research Team and Reflexivity**

Interviews were conducted by J.E., a female PhD student in biomedical ethics. The researchers have backgrounds in Bioethics, Medicine/ Medical Genetics, Political Science, Communication Studies and Theology, and most of them have long-standing experience with qualitative research. One of the interview partners who also co-supervises this project, is a co-author of this paper. No relationship existed between the other study participants and the interviewer prior to this study. Our study seeks to gain a better understanding of the challenges regarding informed consent in paediatric genome-wide sequencing in order to promote patient-centeredness and reduce moral distress of clinicians involved, keeping in mind the limits of the medical system. We are aware that our perspective on informed consent is shaped by the European context, where individual autonomy is a broadly embraced societal value.

Within this exploratory research, an epistemological stance of critical realism is suitable. We are convinced that themes “are actively created by the researcher at the intersection of data, analytic process and subjectivity” (2,p594). Accordingly, our analysis does not pretend to uncover the objective truth that slumbers in the data. There is an observed social reality in the interviews relating to experiences of medical geneticists, but this social reality necessarily includes the interviewer. The themes we developed are our interpretations of the data and are based on our positionality. Therefore, special attention was paid to reflexivity and rigour in the research process in line with the standards set in the consolidated criteria for reporting qualitative research (COREQ). The theoretical frameworks underpinning our study are empirical bioethics (3), person-centred and shared decision-making as well as related bioethical principles and concepts such as informed consent.

### Study Design

Semi-structured interviews with medical geneticists working with children in Switzerland or Germany were conducted. In line with the exploratory nature of the qualitative research strategy, we employed purposive sampling combined with snowball sampling to facilitate access to the participant group (4, 5). We included medical geneticists from two major continental European countries (Germany and Switzerland). In these countries medical geneticists are board certified physicians. The two countries have similar legal and practical conditions. In both countries medical geneticists are typically responsible for obtaining IC for paediatric GWS (genetic counsellors, for example, are not yet a recognized profession). Medical geneticists were identified via the homepages of relevant hospitals and private institutions, as well as via personal contacts and recommendations of the medical geneticists already identified. Electronic study invitation letters were sent to them. One reminder e-invitation letters were sent to non-responders 2-4 weeks later. For all who agreed to participate, a telephone or in-person meeting was scheduled based on medical geneticists’ preferences.

Paediatric healthcare institutions in Germany and Switzerland typically provide care for children and adolescents until they reach 18 years of age. Our interviews focused on cases when parents are taking the decisions as the legal representatives. We interviewed 20 medical geneticists who work with children in Germany (n=10) and in Switzerland (n=10) (German-speaking part and Romandy); 15 worked in academic hospitals and 5 in private specialty practices or private laboratories. These numbers are justified by feasibility within the research project and are a commonly accepted size in qualitative research (6). We adopted a pragmatic approach in determining data saturation and critically evaluated theme saturation throughout the data analysis phase (7). To be considered in our study potential participants needed to be medical geneticists in Germany or Switzerland who are involved in paediatric genome-wide sequencing. Under the current legal and practical circumstances in Germany and Switzerland medical geneticist are from the expert side the group mostly involved in the decision-making process, genetic counselling and know best about the techniques and procedures.

Interviews were conducted between February 2020 and April 2021, in German or English. Only the medical geneticists and the researcher were present during the interview. An interview guideline was developed based on available literature (Supplement) and was piloted in the first 2 interviews. As no problems were identified, no adaptation was deemed to be necessary. All interviews were audio-recorded and had a mean duration of 55 minutes (range 29-71 minutes). They were transcribed verbatim and personal as well as local names were encrypted upon transcription. Transcripts were not returned to medical geneticists.

The research tool of interviewing was chosen, because this method enables access to the interior life of people, gaining an awareness of how they feel, think, interpret and behave – also due to the possibility to ask follow-up questions and to encourage the respondents to develop some of their aspects further (8). Thus, qualitative interviewing enables access to data that could not be disclosed by quantitative methods, and is especially suitable for investigations of sensitive topics (9) – such as ethical issues around paediatric genome-wide sequencing. Quantitative interviews provide high precision through standardisation, but this is often at the expense of completeness or profoundness of the answers gained. Reduced standardisation was accepted in our qualitative interviews for the benefit of increased density of the answers obtained, providing a fuller understanding of the medical geneticist’s views (8). Semi-structured, open-ended, qualitative interviews were chosen to cover a broad topic in a comparable, structured way, the structure of open questions defining the areas to be explored, while providing the space to develop further upon issues medical geneticists regard essential (10).

The interview guide explored a wide range of issues, e.g. 1) in which cases medical geneticists would offer genome-wide sequencing of children to parents 2), how they experience the pretest consultation 3) general attitudes towards the ethical challenges and opportunities raised by genome-wide sequencing in paediatrics. It was created to be flexible allowing further exploration of issues which came up during the interview.

### Data analysis

Using the interview transcriptions in their original language, data were analysed inductively by J.E. and I.K. using the qualitative software MAXQDA and employing reflexive thematic analysis (2, 11). A coding system encompassing 133 codes was developed by comparing and discussing individually developed codes, coded segments and writing memos. In an iterative and interpretive process, main themes on the overall topic of IC were generated and critically reflected and discussed with the other co-authors based on analytic reports written by J.E.

**References**

1. Tong A, Sainsbury P, Craig J. Consolidated criteria for reporting qualitative research (COREQ): a 32-item checklist for interviews and focus groups. International Journal for Quality in Health Care. 2007;19(6):349-57.

2. Braun V, Clarke V. Reflecting on reflexive thematic analysis. Qualitative Research in Sport, Exercise and Health. 2019;11(4):589-97.

3. Ives J, Dunn M, Molewijk B, Schildmann J, Bærøe K, Frith L, et al. Standards of practice in empirical bioethics research: towards a consensus. BMC medical ethics. 2018;19(1):68.

4. Palinkas LA, Horwitz SM, Green CA, Wisdom JP, Duan N, Hoagwood K. Purposeful Sampling for Qualitative Data Collection and Analysis in Mixed Method Implementation Research. Administration and Policy in Mental Health and Mental Health Services Research. 2015;42(5):533-44.

5. Marshall MN. Sampling for qualitative research. Family Practice. 1996;13(6):522-6.

6. Mason M. Sample Size and Saturation in PhD Studies Using Qualitative Interviews. Forum Qualitative Sozialforschung / Forum: Qualitative Social Research. 2010;11(3).

7. Low J. A Pragmatic Definition of the Concept of Theoretical Saturation. Sociological Focus. 2019;52(2):131-9.

8. Weiss R. Learning from strangers: The art and method of qualitative interview studies. New York: Simon and Schuster; 1995.

9. Gill P, Stewart K, Treasure E, Chadwick B. Methods of data collection in qualitative research: interviews and focus groups. Br Dent J. 2008;204(6):291-5.

10. Leech BL. Asking Questions: Techniques for Semistructured Interviews. PS: Political Science &amp; Politics. 2002;35(4):665-8.

11. Braun V, Clarke V. Using thematic analysis in psychology. Qualitative Research in Psychology. 2006;3:77-101.
